# Brain and Body Work

**Published:** 1879-11

**Authors:** 


### BRAIN AND BODY WORK.

Physiologists, after patient and close inquiry, have arrived at the important and practical conclusion that the power of the entire man, his vitality, is as much expended by two hours of deep mental effort as by a whole day of ordinary bodily labor; this fact seems to be founded on observed physiological laws; hence, the man who spends four hours in the twenty-four in earnest mental labor, goes to the utmost allowable limit for a day's work, and all the time that remains, after deducting ten hours for eating, sleeping and dressing, should be conscientiously expended in muscular exercises which require no special brain effort, and such exercises should always, by preference, be those which are agreeable, useful and profitable; for they not only promote the healthful condition of the body, but give rest to the brain, which, by that rest, recuperates its powers ; many can remember, when turning back to their school-days, that they have gone to bed feeling that they did not know their lessons, yet, on rising in the morning, the mind would run over them with a gratifying and surprising clearness. It is this which accounts for the observation that persons have striven hard to remember some important fact, or as to where valuable papers have been laid, and towards morning, when the mind began to awake, a little before the body, this being the time of dreams, the point is made clear in the form of a dream, thus showing that rest of the brain, whether by actual sleep or the passive, comparative rest which manual labor affords, gives mental activity, vigor, perspicacity; from these it follows that no form of muscular exercise is ignoble in a student, a brain-worker, which has to be done by some one, and by being done by him, will save money, or will save the time of another, who, perhaps, may already be over-taxed. How many servants are over-taxed! how many faithful, uncomplaining wives are over-taxed! and sons and daughters sometimes; and clerks, and apprentices, and other employees. In every dwelling in a large city, there are many things which the master could do which would reflect benefit on himself and others also; some of these may be suggested : get up by daylight, clear the snow from the sidewalks, kindle two or three fires, ventilate your parlors, keep the cellar well swept, split up kindling-wood, after sawing it yourself; whitewash the cellar twice a year, as also the fencing around the back-yard; trim the eight or ten grape vines which you ought to have against the fence ; kill off the worms which infest them in the summer; root out the clover and weeds from your grass plat; keep your hundred feet of flower-borders in perfect order; if you have a library, dust your books, rearrange them so that you may be able to put your hand upon them in the dark if needed; assort your pamphlets and magazines, so that no time may be lost should you want any of them in a hurry; in this way valuable time may be saved on occasions when you have no time to spare; then pump water in your tank for twenty minutes every morning.

rank for twenty minutes every morning ; repair all the broken glass yourself ; learn how to keep all cracks in the plastering filled in with plaster of Paris ; keep your roof well painted ; and you great big lazy hulk you, why might'nt you as well, when all these things are done, and you have any unoccupied time, help your wife darn some of the basket full of stockings ; or sew on the lost buttons of your boys who are at school ; or trim their hair, and sew up the rips in shoes, and cut down the pegs or tacks in the inner soles, which so often do permanent injury to the feet ; keep their skates in order ; have a grindstone of your own and an iron vice, and keep all the knives sharp, and the handles tight, learn how to mend broken china and common delf ; to tack down carpets ; to hang pictures ; to take stains out of marble and wood ; to replace mahogany veneering ; to render chair legs and backs firm ; to keep the tubs and barrels hooped up ; learn how to make good flour paste, and keep some always on hand, ready to mend a torn bank note ; or paste a useful newspaper scrap in some appropriate place ; or have a book for domestic receipts, and when you see one which seems to be valuable, paste it in the book under its proper alphabetical head. What a marvellous help any husband might be to his wife and family in ways like these, and be saving many a dollar besides, instead of lolling about on the sofa or chairs ; or, with feet on table or mantle, leaning back and smoking a filthy pipe or noisome cigar, or sipping the murderous brandy and water, or vulgar "Lager ;" or wasting time in pitiful card playing or childish checkers or chess or back gammon or solitaire or any other useless time-murdering, or mind dwindling occupation ; or take a good long walk after tea, with yourself, wife and children to some profitable lecture ; to some prayer meeting, or other useful assemblage of the good, keeping diligently away from the theatre, the dance and the club house, all three, the equal destroyers of social purity, of domestic happiness and family elevation. Fathers, mothers, husbands, wives, think of these things, and be encouraged to do them, by the reflection that the editor of Hall's Journal of Health has been practising thus for many years and keeps young and thrives upon the same in physical well-being and is as lively as a cricket

